# Obesity may increase survival, regardless of nutritional status: a Swedish cohort study in nursing homes

**DOI:** 10.1186/s12877-022-03356-1

**Published:** 2022-08-10

**Authors:** Maria Burman, Carl Hörnsten, Yngve Gustafson, Birgitta Olofsson, Peter Nordström

**Affiliations:** 1 Department of Community Medicine and Rehabilitation, Geriatric Medicine, Umeå University, SE-901 87 Umeå, Sweden; 2 Department of Nursing, Umeå University, Umeå, Sweden

**Keywords:** Obesity, Nutritional status, Mortality, Older nursing home residents

## Abstract

**Background:**

To investigate the associations between the body mass index (BMI), Mini Nutritional Assessment–Short Form (MNA-SF) scores, and 2-year mortality.

**Methods:**

A nationwide cohort study using data from a national quality register of older (age ≥ 65 years) nursing home residents (*N* = 47,686). Individuals were categorized according to BMI as underweight (< 18.5 kg/m^2^), normal-weight (18.5–24.9 kg/m^2^), overweight (25.0–29.9 kg/m^2^), and obese (class I, 30.0–34.9 kg/m^2^; class II, 35.0–39.9 kg/m^2^; class III, ≥ 40.0 kg/m^2^). Participants’ nutritional status were categorized as good (MNA-SF score 12–14), at risk of malnutrition (MNA-SF score 8–11), or malnutrition (MNA-SF score 0–7). Associations with mortality were analysed using Cox proportional-hazards models.

**Results:**

At baseline, 16.0% had obesity, and 14.6% were malnourished. During 2 years of follow-up, 23,335 (48.9%) individuals died. Compared with normal-weight individuals, mortality was greater among underweight individuals [hazard ratio (HR) 1.62, 95% confidence interval (CI) 1.55–1.69] and lesser among individuals with class I (HR 0.63, 95% CI 0.60–0.66), class II (HR 0.62, 95% CI 0.56–0.68), and class III (HR 0.80, 95% CI 0.69–0.94) obesity. Compared with individuals with good nutritional status, mortality was increased for those with malnutrition (HR 2.98,95% CI 2.87–3.10). Lower mortality among obese individuals was also seen in subgroups defined according to MNA-SF scores.

**Conclusions:**

Among older nursing home residents, obesity, including severe obesity, was associated with lower 2-year mortality. Higher BMIs were associated with better survival, regardless of nutritional status according to MNA-SF.

**Supplementary Information:**

The online version contains supplementary material available at 10.1186/s12877-022-03356-1.

## Introduction

The prevalence of obesity is increasing among older adults [1, 2], and was present in 14% of older European nursing home residents [3]. Obesity has several adverse health-related outcomes in older adults [4]. Furthermore, obesity is a well-established risk factor for many diseases, including cardiovascular disease (CVD) [5], cancer [6], and type 2 diabetes mellitus [7], and for death [8–10]. However, the latter association is not straightforward; obesity has been associated with reduced mortality in patients with established CVD (e.g., heart failure and coronary heart disease) [11], chronic obstructive pulmonary disease (COPD) [12], and cancer [13]. This phenomenon has been called the obesity paradox. In older nursing home residents, overweight and obesity have been associated with lower mortality compared to normal-weight [3, 14], and lower mortality has been observed in both shorter and long-term follow-up for obese individuals [15]. Obesity is often defined as a Body Mass Index (BMI [kg/m^2^]) ≥ 30.0 kg/m^2^ and less is known about the association between obesity class I-III and mortality in the population of older nursing home residents. In a study of Grabowski et al. the association between obesity class I-III and mortality showed inconsistent results among older adults in nursing homes. They reported no significant associations between obesity and mortality in the whole sample, however, those newly admitted with obesity class II-III or obesity class III had a higher risk of mortality, whereas lower risk of mortality was found for a BMI > 28 kg/m^2^ in those already living in the nursing homes [16].

On the other side of the spectrum, underweight has been associated with higher mortality in many previous studies [3, 14]. Underweight is often caused by malnutrition, which is common [17–19], and associated with increased mortality [17, 20–22] in older nursing home residents. However, the relationship between body weight and nutritional status is complicated. For example obesity is a risk factor for several diseases and conditions that negatively affect nutritional status, thus obesity can co-exist with malnutrition and/or sarcopenia [23]. Furthermore, weight loss despite a normal/high BMI can indicate malnutrition. Meanwhile it is possible to be underweight and not malnourished [24]. Considering the complicated association between BMI and nutritional status, it may be important to study both their individual and aggregate associations with mortality to better understand nursing home mortality.

Consequently, it is arguably useful to investigate the association between BMI and mortality in a large sample that makes it possible to compare the different obesity classes, and to also investigate the combined effects of BMI and nutritional status, in a population of older nursing home residents. The demographic shift, leading to a larger population of older adults in the future [25, 26], increase the need for further research of these important health issues, especially in this population of frail older nursing home residents. Therefore, the aim of this large nationwide cohort study was to investigate the association between obesity and mortality, including the potential heterogeneity for obesity class I-III. Furthermore, the combined effect of BMI and nutritional status according to the Mini Nutritional Assessment–Short Form (MNA-SF) among older nursing home residents in Sweden was investigated.

## Methods

### Study design and setting

This was a nationwide cohort study based on data from the National quality register Senior Alert (SA) that contains data on older adults who have contact with the healthcare system, most commonly in nursing homes, hospitals and via home care services. Senior Alert has been described in greater detail previously, in summary, several risk assessments, including a risk assessment for malnutrition, are performed and findings are recorded in the register as part of preventive care in which those at malnutrition risk should be further assessed and preventive action plans should be planned and later evaluated [27, 28].

### Inclusion and exclusion criteria

Eligible participants were all nursing home residents aged ≥ 65 years who were registered in SA between 1 January 2012 and 31 December 2013 and who did not fulfil exclusion criteria with missing BMI or MNA-SF data and those with weights < 20 kg or > 210 kg, heights < 100 cm or > 210 cm, BMIs < 10 kg/m^2^ or > 70 kg/m^2^, or documented death before SA registration. The date of inclusion was the date of SA registration, and participants were followed until death or for a maximum of 2 years. Caregivers were obliged to inform all persons assessed about their SA registration and all persons registered were able to withdraw from the register (opt-out) at any point [27]. This study was conducted in accordance with the principles of the Declaration of Helsinki. The study was approved by the Regional Ethical Review Board in Umeå (2013–86-31 M and 2013–456-32 M).

### Participants’ characteristics

In SA, the risk of malnutrition was assessed with the six-item MNA-SF [29, 30], which covers respondents’ decline in food intake, unintentional weight loss in the past 3 months, mobility, psychological stress and/or acute disease in the past 3 months, neuropsychological problems (dementia or depression), and BMI (BMI; < 19 kg/m^2^, 0 points; 19 to < 21 kg/m^2^, 1 point; 21 to < 23 kg/m^2^, 2 points; ≥ 23 kg/m^2^, 3 points). The sum of these items’ score gives the total screening score. The maximum possible MNA-SF score is 14; scores of 0–7 indicate malnutrition, scores of 8–11 indicate the risk of malnutrition, and scores of 12–14 indicate good nutritional status [29, 30]. Body Mass Index is also registered in SA and according to the World Health Organization’s (WHO) categories, participants’ BMIs were used to classify them as individuals with underweight (< 18.5 kg/m^2^), normal-weight (18.5–24.9 kg/m^2^), overweight (25.0–29.9 kg/m^2^), and obesity (class I, 30.0–34.9 kg/m^2^; class II, 35.0–39.9 kg/m^2^; class III, ≥ 40.0 kg/m^2^) [31]. Information about participants’ diagnoses (dementia, hip fracture, COPD, renal failure, rheumatoid arthritis, myocardial infarction, stroke, and diabetes) was collected from the National Patient Register (NPR), which has full national coverage of patients discharged from hospitals since 1987. In 2001, the registry of diagnoses made during outpatient specialized care was introduced [32, 33]. Diagnoses are coded in the NPR according to the Swedish version of the International Classification of Diseases and Related Health Problems, Tenth Revision [34, 35]. Prevalent diagnoses were determined based on diagnosis occurrence in the registry up to the date of inclusion. Information about participants’ level of education and disposable income was collected from Statistics Sweden, a government agency providing official statistics in Sweden. Mortality data were collected from the Cause of Death Register, which has full national coverage since 1961 [36].

### Statistical analyses

Analyses of potential differences in sex and age between participants and non-participants were performed using the chi-squared test and the independent-samples *t* test, respectively. For comparisons among participant groups, defined by MNA-SF categories, the chi-squared test was used for categorical variables and the Welch one-way analysis of variance test was used for continuous variables. Associations between BMI, MNA-SF scores, and mortality were explored using Cox proportional-hazards models, including the combined effect of BMI and MNA on mortality. The regression of Schoenfeld residuals was used to test the proportional hazards assumption for all included variables against time. As the BMI and MNA-SF data violated this assumption, the observation period was divided into intervals (0 to < 6 months, 6 to < 12 months, 12 to < 18 months, 18–24 months, and 0–24 months) and separate regression analyses were performed. All models were adjusted for age, sex, level of education, disposable income, dementia, hip fracture, COPD, renal failure, rheumatoid arthritis, myocardial infarction, stroke, and diabetes, and variables that violated the proportional hazards assumption were included by stratification (except BMI and MNA-SF). Kaplan–Meier survival curves were used to visualize the associations between BMI, MNA-SF scores, and mortality. Separate analyses were performed for obese participants overall (BMI ≥30.0 kg/m^2^) and by obesity classes. Subgroup analyses were performed to explore differences between women and men, and these were adjusted for age, level of education, disposable income, dementia, hip fracture, COPD, renal failure, rheumatoid arthritis, myocardial infarction, stroke, and diabetes. To investigate the interaction with sex, likelihood ratio tests were performed of models with the interaction term (BMI(continuous)/BMI(categorized)/MNA(continuous)/MNA(categorized) × sex) and models without interaction terms. *P* values < 0.05 were considered to be significant. The statistical analyses were using R (version 3.5.0, The R Foundation for Statistical Computing, Vienna, Austria).

## Results

Of 49,604 individuals identified in the SA registry, the cumulative dropouts were; death before SA registration (*n* = 273), missing MNA-SF (*n* = 1612), weight < 20 kg (*n* = 1), missing weight (*n* = 17), height < 100 cm (*n* = 11), height > 210 cm (*n* = 1), BMI < 10 kg/m^2^ (*n* = 2) and BMI > 70 kg/m^2^ (*n* = 1). In total, 1918 individuals were excluded resulting in a final sample of 47,686 individuals (70% women) with a mean age of 86.3 ± 7.4 years. Excluded individuals were older than participants (86.8 ± 7.3 years, *p* = 0.006), with no difference in sex distribution (69.6% women).

About one-third of participants were diagnosed with dementia, 21.8% had had hip fractures, and 22.6% had had strokes at baseline. Overall, 8.3% of participants were underweight, 30.7% were overweight, and 16.0% were obese (12.1, 3.0, 0.9% had obesity class I, II, III, respectively). According to MNA-SF scores, 14.6% of participants were malnourished and 45.0% were at risk of malnutrition; 15.0% of the individuals with malnutrition were overweight or obese. During the 2-year follow-up period, 23,335 (48.9%) individuals died and 31.7% of these deaths occurred during the first 6 months, resulting in a mean follow up time of 1.4 years. The last documented death in the cohort occurred on 20 March 2015. Most participant characteristics differed among MNA-SF categories (Table 1).

**Table 1:** Baseline characteristics of participants

	Whole sample	MNA-SF 0–7	MNA-SF 8–11	MNA-SF 12–14	*p*
Number	47,686	6970	21,459	19,257	< 0.001
Age, mean (SD)	86.3 (7.4)	86.7 (7.2)	86.3 (7.4)	86.3 (7.4)	< 0.001
Women, n (%)	33,374 (70.0)	5121 (73.5)	15,231 (71.0)	13,022 (67.6)	< 0.001
Level of education, n (%)					< 0.001
< 9 years	26,534 (57.1)	3666 (54.2)	11,811 (56.5)	11,057 (58.9)	
9–12 years	15,744 (33.9)	2435 (36.0)	7104 (34.0)	6205 (33.0)	
> 12 years	4159 (9.0)	669 (9.9)	1975 (9.5)	1515 (8.1)	
Income^a^, mean (SD)	167 (202)	164 (188)	165 (193)	170 (217)	0.013
Dementia, n (%)	15,193 (31.9)	2948 (42.3)	7628 (35.5)	4617 (24.0)	< 0.001
Hip fracture, n (%)	10,399 (21.8)	2066 (29.6)	4992 (23.3)	3341 (17.3)	< 0.001
COPD, n (%)	2567 (5.4)	438 (6.3)	1142 (5.3)	987 (5.1)	0.001
Renal failure, n (%)	1227 (2.6)	180 (2.6)	532 (2.5)	515 (2.7)	0.46
Rheumatoid arthritis, n (%)	1059 (2.2)	161 (2.3)	509 (2.4)	389 (2.0)	0.048
Myocardial infarction, n (%)	6460 (13.5)	874 (12.5)	2799 (13.0)	2787 (14.5)	< 0.001
Stroke, n (%)	10,764 (22.6)	1604 (23.0)	5016 (23.4)	4144 (21.5)	< 0.001
Diabetes mellitus, n (%)	7765 (16.3)	989 (14.2)	3336 (15.5)	3440 (17.9)	< 0.001
BMI, mean (SD)	25.1 (5.2)	20.8 (4.5)	24.4 (4.9)	27.4 (4.5)	< 0.001
BMI categories, n (%)					< 0.001
< 18.5	3959 (8.3)	2288 (32.8)	1671 (7.8)	0 (0.0)	
18.5–24.9	21,445 (45.0)	3641 (52.2)	11,385 (53.1)	6419 (33.3)	
25.0–29.9	14,655 (30.7)	750 (10.8)	5728 (26.7)	8177 (42.5)	
≥ 30.0	7627 (16.0)	291 (4.2)	2675 (12.5)	4661 (24.2)	
BMI obesity class I-III, n (%)					< 0.001^b^
30.0–34.9	5774 (12.1)	225 (3.2)	2034 (9.5)	3515 (18.3)	
35.0–39.9	1419 (3.0)	46 (0.7)	493 (2.3)	880 (4.6)	
≥ 40.0	434 (0.9)	20 (0.3)	148 (0.7)	266 (1.4)	
MNA-SF, mean (SD)	10.4 (2.6)	5.6 (1.5)	9.8 (1.1)	12.8 (0.8)	< 0.001

Differences between groups (defined by MNA-SF categories) were analysed using chi-squared test for categorical variables and using Welch one-way analysis of variance tests for continuous variables. *BMI* Body mass index (kg/m^2^), *COPD* Chronic obstructive pulmonary disease, *MNA-SF* Mini Nutritional Assessment-Short Form, *SD* Standard deviation. ^a^Disposable income, in 1000 SEK per year. ^b^Analyses of all BMI categories with obesity divided in class I, II, and III

**Fig. 1:**
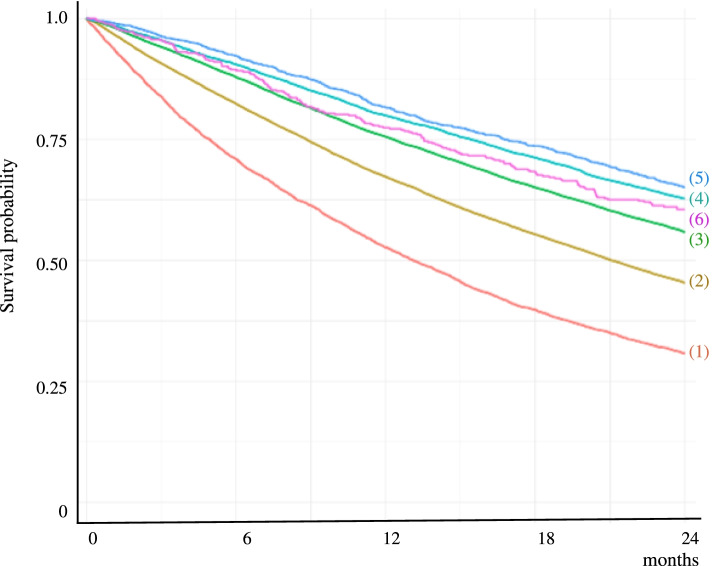
Kaplan–Meier survival curves showing the association between body mass index (BMI) and 2-year mortality. (1) = BMI < 18.5 kg/m^2^; (2) = BMI 18.5–24.9 kg/m^2^; (3) = BMI 25.0–29.9 kg/m^2^; (4) = BMI 30.0–34.9 kg/m^2^; (5) = BMI 35.0–39.9 kg/m^2^; (6) = BMI ≥ 40.0 kg/m^2^

**Fig. 2:**
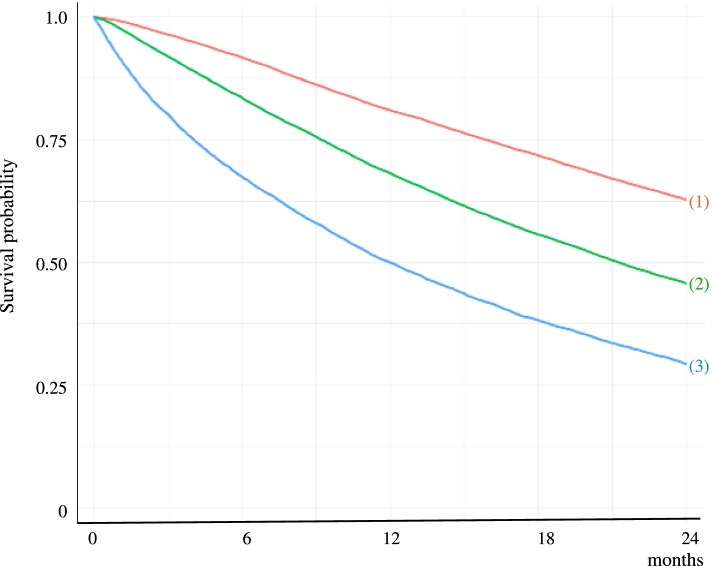
Kaplan–Meier survival curves showing the association between Mini Nutritional Assessment–Short Form (MNA-SF) scores and 2-year mortality. (1) = MNA-SF 12–14; (2) = MNA-SF 8–11; (3) = MNA-SF 0–7

Kaplan–Meier survival curves show the associations of 2-year mortality with BMI (Fig. 1) and MNA-SF scores (Fig. 2). Adjusted associations are presented in Table 2 (unadjusted results, which were similar, are presented in the Appendix, Table A1). Compared with normal-weight individuals, 2-year mortality was greater among underweight individuals [hazard ratio (HR) 1.62, 95% confidence interval (CI) 1.55–1.69], and lesser among overweight individuals (HR 0.74, 95% CI 0.72–0.76) and those with class I (HR 0.63, 95% CI 0.60–0.66), class II (HR 0.62, 95% CI 0.56–0.68), and class III (HR 0.80, 95% CI 0.69–0.94) obesity. In the analyses with obesity defined as BMI ≥ 30.0 kg/m^2^, mortality was lower among obese individuals than among overweight (HR 1.17, 95% CI 1.12–1.22), normal-weight (HR 1.58, 95% CI 1.51–1.65), and underweight (HR 2.56, 95% CI 2.42–2.71) individuals. Compared with individuals with good nutritional status according to MNA-SF, mortality was greater among individuals at risk of malnutrition (HR 1.74, 95% CI 1.69–1.79) and those with malnutrition (HR 2.98, 95% CI 2.87–3.10). The associations of mortality with BMI and MNA-SF scores persisted throughout all follow-up periods, except for obesity class III where lower mortality was found in analyses of the entire study period of 24 months and in the first 6 months of follow-up (Table 2).

**Table 2:** Adjusted Cox proportional hazards for associations of BMI and MNA-SF scores with 2-year all-cause mortality during follow-up intervals

		Hazard Ratio (95% Confidence Interval)				
		0–24 months	0–6 months	6–12 months	12–18 months	18–24 months
	Deaths n(%)	(*n* = 47,686)	(*n* = 47,686)	(*n* = 40,283)	(*n* = 33,704)	(*n* = 27,190)
Deaths, n (%)	< 0.001^a^	23,335 (48.9)	7403 (15.5)	6579 (16.3)	5209 (15.5)	4144 (15.2)
BMI categ. (kg/m^2^)	*p* < 0.001^b^					
≥ 30.0	2757 (36.1)	1	1	1	1	1
25.0–29.9	6354 (43.4)	1.17 (1.12–1.22)	1.24 (1.13–1.36)	1.12 (1.03–1.22)	1.19 (1.09–1.31)	1.13 (1.02–1.25)
18.5–24.9	11,519 (53.7)	1.58 (1.51–1.65)	1.84 (1.70–2.01)	1.48 (1.37–1.61)	1.52 (1.39–1.67)	1.44 (1.31–1.59)
< 18.5	2705 (68.3)	2.56 (2.42–2.71)	3.45 (3.13–3.80)	2.26 (2.03–2.51)	2.31 (2.04–2.60)	1.92 (1.67–2.21)
BMI categ. (kg/m^2^)	*p* < 0.001^b^					
18.5–24.9	11,519 (53.7)	1	1	1	1	1
< 18.5	2705 (68.3)	1.62 (1.55–1.69)	1.87 (1.75–2.00)	1.52 (1.40–1.66)	1.51 (1.37–1.67)	1.33 (1.18–1.50)
25.0–29.9	6354 (43.4)	0.74 (0.72–0.76)	0.67 (0.63–0.71)	0.75 (0.71–0.80)	0.78 (0.73–0.84)	0.79 (0.73–0.84)
30.0–34.9	2106 (36.5)	0.63 (0.60–0.66)	0.55 (0.50–0.60)	0.66 (0.60–0.72)	0.65 (0.59–0.72)	0.69 (0.62–0.77)
35.0–39.9	482 (34.0)	0.62 (0.56–0.68)	0.48 (0.39–0.58)	0.70 (0.59–0.83)	0.64 (0.53–0.77)	0.69 (0.56–0.84)
≥ 40.0	169 (38.9)	0.80 (0.69–0.94)	0.71 (0.53–0.96)	0.87 (0.65–1.15)	0.83 (0.60–1.15)	0.82 (0.57–1.17)
MNA-SF categ.	*p* < 0.001^b^					
12–14	7023 (36.5)	1	1	1	1	1
8–11	11,444 (53.3)	1.74 (1.69–1.79)	2.10 (1.97–2.23)	1.68 (1.59–1.78)	1.72 (1.62–1.83)	1.48 (1.38–1.58)
0–7	4868 (69.8)	2.98 (2.87–3.10)	4.78 (4.48–5.11)	2.50 (2.32–2.69)	2.33 (2.14–2.54)	2.01 (1.82–2.22)

Analyses were of MNA-SF scores (ref. 12–14), BMIs with obesity defined as BMI ≥ 30.0 kg/m^2^ (ref.) and BMIs with obesity divided into class I, II, III (ref. 18.5–24.9 kg/m^2^). They were adjusted for age, sex, education level, disposable income, dementia, hip fracture, chronic obstructive pulmonary disease, renal failure, rheumatoid arthritis, myocardial infarction, stroke and diabetes. *BMI* Body mass index (kg/m^2^), *MNA-SF* Mini Nutritional Assessment–Short Form. ^a^ *P* value for number of deaths in follow-up intervals. ^b^*P* values for numbers of deaths in BMI categories and MNA-SF categories, respectively

Adjusted associations between BMI and 2-year mortality according to MNA-SF categories are presented in Table 3 (unadjusted results, which were similar, are presented in the Appendix, Table A2). Compared with individuals with obesity and good nutritional status according to MNA-SF scores, the mortality rate was greater among overweight, and normal-weight individuals with MNA-SF scores 12–14. Also compared to obese individuals with good nutritional status, greater mortality was found in those at risk of malnutrition (MNA-SF scores of 8–11) irrespective of BMI categories, with an increasing trend seen with lower BMIs. Greater mortality was found with all BMI categories among individuals with malnutrition according to MNA-SF scores, and especially in those with underweight according to BMI. The same pattern was seen in all follow-up periods except among individuals with a good nutritional status according to MNA-SF scores and overweight where some of the follow-up periods did not reach significance (Table 3). Adjusted and unadjusted subgroup analyses revealed slightly greater mortality among men with low MNA-SF scores and BMIs compared with their female counterparts (in the Appendices, Table A3 and A4, respectively).

**Table 3:** Adjusted Cox proportional hazards for the association of BMI according to MNA-SF score with 2-year all-cause mortality

			Hazard Ratio (95% Confidence Interval)				
	n	Deaths n(%)	0–24 months	0–6 months	6–12 months	12–18 months	18–24 months
**MNA-SF 12–14**							
BMI ≥30.0	4661	1409 (30.2)	1	1	1	1	1
BMI 25.0–29.9	8177	2969 (36.3)	1.13 (1.06–1.20)	1.09 (0.95–1.25)	1.11 (0.98–1.25)	1.11 (0.98–1.27)	1.22 (1.06–1.39)
BMI 18.5–24.9	6419	2645 (41.2)	1.28 (1.20–1.37)	1.36 (1.18–1.56)	1.22 (1.08–1.38)	1.22 (1.07–1.40)	1.38 (1.20–1.58)
BMI < 18.5	0	NA	NA	NA	NA	NA	NA
**MNA-SF 8–11**							
BMI ≥ 30.0	2675	1168 (43.7)	1.60 (1.48–1.73)	1.78 (1.52–2.09)	1.61 (1.39–1.86)	1.42 (1.21–1.67)	1.62 (1.37–1.92)
BMI 25.0–29.9	5728	2887 (50.4)	1.86 (1.74–1.98)	2.30 (2.01–2.62)	1.72 (1.52–1.93)	1.84 (1.62–2.10)	1.61 (1.39–1.86)
BMI 18.5–24.9	11,385	6378 (56.0)	2.12 (2.00–2.25)	2.59 (2.29–2.93)	2.00 (1.79–2.23)	2.04 (1.81–2.30)	1.92 (1.69–2.19)
BMI < 18.5	1671	1011 (60.5)	2.42 (2.23–2.63)	2.92 (2.48–3.42)	2.22 (1.90–2.59)	2.56 (2.17–3.03)	2.06 (1.69–2.51)
**MNA-SF 0–7**							
BMI ≥ 30.0	291	180 (61.9)	2.86 (2.44–3.36)	4.81 (3.74–6.17)	1.85 (1.31–2.61)	2.66 (1.89–3.74)	2.20 (1.44–3.37)
BMI 25.0–29.9	750	498 (66.4)	3.17 (2.86–3.52)	5.48 (4.62–6.51)	2.29 (1.86–2.84)	2.36 (1.86–3.01)	2.40 (1.83–3.14)
BMI 18.5–24.9	3641	2496 (68.6)	3.25 (3.03–3.47)	5.14 (4.52–5.85)	2.71 (2.38–3.07)	2.54 (2.20–2.93)	2.38 (2.02–2.79)
BMI < 18.5	2288	1694 (74.0)	4.01 (3.73–4.32)	6.47 (5.65–7.40)	3.36 (2.92–3.86)	2.92 (2.47–3.44)	2.69 (2.22–3.26)

The reference group was MNA-SF score = 12–14 and BMI ≥ 30.0 kg/m^2^. Analyses were adjusted for age, sex, education level, disposable income, dementia, hip fracture, chronic obstructive pulmonary disease, renal failure, rheumatoid arthritis, myocardial infarction, stroke and diabetes. BMI, body mass index (kg/m^2^); *MNA-SF* Mini Nutritional Assessment–Short Form, *NA* Not available

## Discussion

In this nationwide cohort study of older nursing home residents in Sweden, overweight and obesity according to the BMI, and malnutrition and the risk thereof according to MNA-SF were common. During the 2-year follow-up period, about half of the population died. Underweight according to the BMI and malnutrition according to MNA-SF were associated independently with greater mortality, compared to normal-weight and MNA-SF score indicating good nutritional status, respectively. Overweight and obesity were associated with lower mortality than was normal-weight. Obesity was associated with reduced mortality, irrespective of MNA-SF scores.

The lower mortality rates observed for obese individuals and those with good nutritional status are in agreement with previous findings [3, 14, 20, 21]. The associations appeared to be stronger during the first 6 months of follow-up, which might be explained by the protection that a larger energy reserve offers during acute illness, in fact, deteriorating health is one of the situations where risk assessments and SA registration is recommended. Researchers have suggested that the main concern for older adults and people with chronic diseases is wasting disease, rather than traditional CVD risk factors [37], and that longer follow-up periods are needed to observe the negative effects of obesity [38]. However, the lower mortality among obese individuals and those with good nutritional status according to MNA-SF persisted throughout the follow-up period in this study, reducing the potential for reverse causality. Hence, in this study cohort, obesity and good nutritional status according to MNA-SF were favourable in the short and long terms.

In the literature, obesity is often defined as a BMI ≥ 30.0 kg/m^2^, which has been associated with lower mortality compared to normal-weight among nursing home residents [3, 14]. Using the subdivision for obesity, lower mortality has been found with class I obesity while results for obesity class II and III has been inconclusive in studies of older adults in the general population [39, 40]. Grabowski et al. [16] reported, in a study conducted in 1996, higher mortality for obesity class II and III in newly admitted nursing home residents while lower mortality was seen for BMI > 28 kg/m^2^ in those who were already residing in the nursing homes. The present study did not have information on when the SA registration was made, or if any preventive actions were planned and their potential effect. It is possible that the care preventive process might have had an effect on the associations with mortality. None the less, the present findings suggest a survival benefit among older nursing home residents with obesity, including class I, II, and III.

To the best of our knowledge, it has not been shown before that obesity reduces mortality risk independently of nutritional status according to MNA-SF. We can only speculate about the factors underlying the observed associations, as the obesity paradox is not fully understood and many contributing factors have been proposed [38, 41]. Higher BMI reflect a protective energy reserve and indicate a preserved muscle mass in a state of malnutrition. Also, older, obese nursing home residents are a selected group of survivors [38]. Furthermore, obesity can contribute to a decline in nutritional state [23]. The present study results do contribute to the complex field of obesity and malnutrition and indicate that higher BMI regardless of the assessed nutritional status according to MNA-SF is protective.

### Study limitations and strengths

This study has some limitations that should be addressed. As it was observational, causality could not be assessed. In addition, the BMI is a limited measure [42]; it does not provide information about muscle mass, body fat distribution, or cardiorespiratory fitness [38]. It is however, a well-established and accessible measure [31]. Moreover, a small proportion (0.9%) of participants in this study had class III obesity, which limited the ability to draw conclusions for this group. Furthermore, the BMI is included in the MNA-SF score; thus these variables are not independent of each other, which must be considered when interpreting the results. Dementia, hip fracture, COPD, and stroke are expected to be more common among those classified as malnourished according to MNA-SF scores as they affect items of the MNA-SF questionnaire directly as a diagnosis or indirectly by affecting food intake, mobility, weight and/or BMI. These diagnoses are also associated with increased mortality and could thus affect the associations of mortality with the BMI and MNA-SF score. Also, there are other diagnoses and conditions than those available for this study, that might affect nutritional status and BMI. Finally, the BMI and MNA-SF data violated the proportional hazards assumption in this study, which was addressed by performing separate analyses for follow-up intervals.

To our knowledge, this study is the largest to examine associations of the BMI, and MNA-SF score with mortality among older nursing home residents and due to the inclusion criteria has a good representativity and generalizability of the findings. Almost half of the population died during the 2-year follow-up period, in agreement with findings from previous studies conducted in Sweden and Europe [3, 17]. In addition, the prevalence of underweight, overweight, and obesity according to the BMI, and malnutrition, and the risk of malnutrition according to MNA-SF were in agreement with previously reported values [3, 17]. Furthermore, the prevalence of dementia, and higher prevalence of dementia among malnourished individuals, concurs with findings from previous studies of older adults in nursing homes in Sweden [17]. Thus, the study cohort seemed to be representative of older adults in nursing homes, thus increasing the generalizability of the present findings.

## Conclusions

This large study of older adults in nursing homes revealed greater mortality among underweight and malnourished individuals according to the BMI and MNA-SF, respectively. Furthermore, overweight and obesity were associated with lower mortality, and higher BMIs, indicating preserved muscle mass, were associated with lower mortality, regardless of nutritional status according to MNA-SF. This study adds to the understanding on the associations among BMI, nutritional status and mortality. Future research investigating underlying factors for these associations would be valuable.

## Supplementary Information

**Additional file 1: Appendix Table A1.** Unadjusted Cox proportional hazards for associations of BMI and MNA-SF scores with 2-year all-cause mortality during follow-up intervals. **Appendix Table A2.** Unadjusted Cox proportional hazards for the association of BMI according to MNA-SF score with 2-year all-cause mortality. **Appendix Table A3.** Adjusted Cox proportional hazards for associations of BMI and MNA-SF scores with 2-year all-cause mortality, in women and men. **Appendix Table A4.** Unadjusted Cox proportional hazards for associations of BMI and MNA-SF scores with 2-year all-cause mortality, in women and men.

## Data Availability

The datasets generated and analysed during the current study are available from the corresponding author on reasonable request.

## Abbreviations

BMI: Body mass index
CI: Confidence interval
COPD: Chronic obstructive pulmonary disease
CVD: Cardiovascular disease
HR: Hazard ratio
MNA–SF: Mini Nutritional Assessment – Short Form
NPR: National Patient Register
SA: Senior Alert
WHO: World Health Organization
