# Supplementary material for: Obesity may increase survival, regardless of nutritional status: a Swedish cohort study in nursing homes
Source: BMC Geriatr. 2022 Aug 10;22:655. doi: 10.1186/s12877-022-03356-1 (PMC9364570; doi:10.1186/s12877-022-03356-1)
Supplement: Supplementary file 1 — Additional file 1: Appendix Table A1. Unadjusted Cox proportional hazards for associations of BMI and MNA-SF scores with 2-year all-cause mortality during follow-up intervals. Appendix Table A2. Unadjusted Cox proportional hazards for the association of BMI according to MNA-SF score with 2-year all-cause mortality. Appendix Table A3. Adjusted Cox proportional hazards for associations of BMI and MNA-SF scores with 2-year all-cause mortality, in women and men. Appendix Table A4. Unadjusted Cox proportional hazards for associations of BMI and MNA-SF scores with 2-year all-cause mortality, in women and men. [file 12877_2022_3356_MOESM1_ESM.docx]

| **Appendix Table A1.** Unadjusted Cox proportional hazards for associations of BMI and MNA-SF scores with 2-year all-cause mortality during follow-up intervals | | | | | |
| --- | --- | --- | --- | --- | --- |
|  | Hazard Ratio (95 % Confidence Interval) | | | | |
|  | 0–24 months | 0–6 months | 6–12 months | 12–18 months | 18–24 months |
|  | (n = 47,686) | (n = 47,686) | (n = 40,283) | (n = 33,704) | (n = 27,190) |
| BMI categ. (kg/m^2^) |  |  |  |  |  |
| ≥30.0 | 1 | 1 | 1 | 1 | 1 |
| 25.0–29.9 | 1.27 (1.21–1.33) | 1.34 (1.23–1.46) | 1.21 (1.11–1.31) | 1.31 (1.20–1.44) | 1.22 (1.10–1.34) |
| 18.5–24.9 | 1.74 (1.67–1.81) | 2.01 (1.86–2.18) | 1.62 (1.50–1.75) | 1.71 (1.57–1.87) | 1.59 (1.45–1.74) |
| <18.5 | 2.71 (2.57–2.86) | 3.59 (3.27–3.94) | 2.38 (2.16–2.64) | 2.48 (2.21–2.78) | 2.07 (1.80–2.36) |
| BMI categ. (kg/m^2^) |  |  |  |  |  |
| 18.5–24.9 | 1 | 1 | 1 | 1 | 1 |
| <18.5 | 1.56 (1.49–1.63) | 1.78 (1.67–1.91) | 1.47 (1.36–1.59) | 1.45 (1.31–1.59) | 1.30 (1.15–1.46) |
| 25.0–29.9 | 0.73 (0.71–0.75) | 0.67 (0.63–0.71) | 0.74 (0.70–0.79) | 0.77 (0.72–0.82) | 0.77 (0.71–0.82) |
| 30.0–34.9 | 0.58 (0.56–0.61) | 0.51 (0.47–0.56) | 0.61 (0.56–0.67) | 0.59 (0.54–0.65) | 0.63 (0.57–0.70) |
| 35.0–39.9 | 0.53 (0.49–0.58) | 0.42 (0.34–0.50) | 0.60 (0.51–0.71) | 0.53 (0.44–0.64) | 0.61 (0.51–0.74) |
| ≥40.0 | 0.64 (0.55–0.74) | 0.58 (0.43–0.77) | 0.72 (0.54–0.94) | 0.64 (0.47–0.88) | 0.62 (0.43–0.87) |
| MNA-SF categ. |  |  |  |  |  |
| 12–14 | 1 | 1 | 1 | 1 | 1 |
| 8–11 | 1.71 (1.66–1.76) | 2.07 (1.95–2.19) | 1.65 (1.56–1.74) | 1.68 (1.58–1.79) | 1.46 (1.37–1.56) |
| 0–7 | 2.89 (2.79–3.00) | 4.64 (4.35–4.95) | 2.43 (2.27–2.61) | 2.23 (2.05–2.42) | 1.97 (1.79–2.17) |
| Analyses were of MNA-SF scores (ref. 12–14), BMIs with obesity defined as BMI ≥ 30.0 kg/m^2^ (ref.) and BMIs with obesity divided into classes I–III (ref. 18.5–24.9 kg/m^2^). BMI, body mass index (kg/m^2^); MNA-SF, Mini Nutritional Assessment–Short Form. | | | | | |

| **Appendix Table A2**. Unadjusted Cox proportional hazards for the association of BMI according to MNA-SF score with 2-year all-cause mortality | | | | | |
| --- | --- | --- | --- | --- | --- |
|  | Hazard Ratio (95 % Confidence Interval) | | | | |
|  | 0−24 months | 0−6 months | 6−12 months | 12−18 months | 18−24 months |
| **MNA-SF 12−14** |  |  |  |  |  |
| BMI ≥30.0 | 1 | 1 | 1 | 1 | 1 |
| BMI 25.0−29.9 | 1.24 (1.17−1.32) | 1.20 (1.04−1.37) | 1.22 (1.08−1.37) | 1.24 (1.10−1.41) | 1.34 (1.18−1.53) |
| BMI 18.5−24.9 | 1.46 (1.37−1.56) | 1.53 (1.33−1.75) | 1.37 (1.22−1.55) | 1.43 (1.26−1.62) | 1.59 (1.39−1.83) |
| BMI <18.5 | NA | NA | NA | NA | NA |
| **MNA-SF 8−11** |  |  |  |  |  |
| BMI ≥30.0 | 1.60 (1.48−1.73) | 1.77 (1.52−2.08) | 1.60 (1.39−1.84) | 1.41 (1.20−1.65) | 1.66 (1.41−1.96) |
| BMI 25.0−29.9 | 1.97 (1.85−2.10) | 2.45 (2.15−2.78) | 1.80 (1.60−2.03) | 1.97 (1.73−2.23) | 1.72 (1.49−1.98) |
| BMI 18.5−24.9 | 2.31 (2.18−2.45) | 2.81 (2.49−3.16) | 2.15 (1.93−2.39) | 2.25 (2.00−2.52) | 2.11 (1.87−2.40) |
| BMI <18.5 | 2.61 (2.41−2.83) | 3.10 (2.66−3.63) | 2.39 (2.06−2.77) | 2.77 (2.36−3.26) | 2.26 (1.86−2.75) |
| **MNA-SF 0–7** |  |  |  |  |  |
| BMI ≥30.0 | 2.87 (2.45−3.35) | 4.81 (3.76−6.14) | 1.90 (1.35−2.65) | 2.53 (1.80−3.55) | 2.30 (1.53−3.46) |
| BMI 25.0−29.9 | 3.28 (2.96−3.63) | 5.73 (4.84−6.79) | 2.37 (1.92−2.93) | 2.41 (1.90−3.06) | 2.50 (1.92−3.25) |
| BMI 18.5−24.9 | 3.47 (3.25−3.70) | 5.47 (4.83−6.20) | 2.89 (2.56−3.27) | 2.71 (2.36−3.12) | 2.55 (2.18−2.98) |
| BMI <18.5 | 4.15 (3.86−4.45) | 6.61 (5.80−7.53) | 3.45 (3.01−3.94) | 3.05 (2.60−3.57) | 2.87 (2.39−3.46) |
| The reference group was MNA-SF score = 12–14 and BMI ≥ 30.0 kg/m^2^. BMI, body mass index (kg/m^2^); MNA-SF, Mini Nutritional Assessment–Short Form; NA, not available. | | | | | |

| **Appendix Table A3**. Adjusted Cox proportional hazards for associations of BMI and MNA-SF scores with 2-year all-cause mortality, in women and men | | | |
| --- | --- | --- | --- |
|  | Hazard Ratio  (95% Confidence Interval) | |  |
|  | Women | Men |  |
|  | (n = 33,374) | (n = 14,312) | ***p*** |
| Deaths n (%) | 15,850 (47.5) | 7485 (52.3) | < 0.001 |
|  |  |  |  |
| BMI continuous (kg/m^2^) | 0.95 (0.95–0.96) | 0.93 (0.92–0.94) | < 0.001* |
| BMI categories (kg/m^2^) |  |  |  |
| ≥30.0 | 1 | 1 | < 0.001* |
| 25.0–29.9 | 1.14 (1.08–1.20) | 1.28 (1.17–1.39) |  |
| 18.5–24.9 | 1.48 (1.40–1.55) | 1.86 (1.71–2.02) |  |
| <18.5 | 2.36 (2.21–2.51) | 3.34 (2.98–3.75) |  |
|  |  |  |  |
| MNA-SF continuous | 0.86 (0.86–0.87) | 0.85 (0.84–0.86) | < 0.001* |
| MNA-SF categories |  |  |  |
| 12–14 | 1 | 1 | < 0.001* |
| 8–11 | 1.75 (1.68–1.81) | 1.72 (1.63–1.81) |  |
| 0–7 | 2.88 (2.75–3.02) | 3.24 (3.03–3.47) |  |
| Analyses were of MNA-SF scores (ref. 12–14), and BMIs with obesity defined as BMI ≥ 30.0 kg/m^2^ (ref.) They were adjusted for age, education level, disposable income, dementia, hip fracture, chronic obstructive pulmonary disease, renal failure, rheumatoid arthritis, myocardial infarction, stroke, and diabetes. * *P* value of the difference between women and men using likelihood ratio tests. BMI, body mass index (kg/m^2^); MNA-SF, Mini Nutritional Assessment-Short Form | | | |

| **Appendix Table A4**. Unadjusted Cox proportional hazards for associations of BMI and MNA-SF scores with 2-year all-cause mortality, in women and men | | | |
| --- | --- | --- | --- |
|  | Hazard Ratio  (95 % Confidence Interval) | |  |
|  | Women | Men |  |
|  | (n = 33,374) | (n = 14,312) | *P* |
| Deaths n (%) | 15,850 (47.5) | 7485 (52.3) | < 0.001 |
|  |  |  |  |
| BMI continuous (kg/m^2^) | 0.95 (0.95–0.95) | 0.93 (0.92–0.93) | < 0.001* |
| BMI categories (kg/m^2^) |  |  |  |
| ≥30.0 | 1 | 1 | < 0.001* |
| 25.0–29.9 | 1.20 (1.14–1.26) | 1.41 (1.30–1.54) |  |
| 18.5–24.9 | 1.60 (1.53–1.68) | 2.09 (1.93–2.26) |  |
| <18.5 | 2.56 (2.41–2.72) | 3.52 (3.15–3.94) |  |
|  |  |  |  |
| MNA-SF continuous | 0.86 (0.86–0.87) | 0.85 (0.85–0.86) | 0.003* |
| MNA-SF categories |  |  |  |
| 12–14 | 1 | 1 | 0.002* |
| 8–11 | 1.74 (1.68–1.80) | 1.68 (1.60–1.77) |  |
| 0–7 | 2.86 (2.74–2.99) | 3.15 (2.95–3.36) |  |
| Analyses were of MNA-SF scores (ref. 12–14), and BMIs with obesity defined as BMI ≥ 30.0 kg/m^2^ (ref.). * *P* value of the difference between women and men using likelihood ratio tests. BMI, body mass index (kg/m^2^); MNA-SF, Mini Nutritional Assessment-Short Form | | | |
